# Supplementary material for: Modeling glioblastoma heterogeneity as a dynamic network of cell states
Source: Mol Syst Biol. 2021 Sep 16;17(9):e10105. doi: 10.15252/msb.202010105 (PMC8444284; doi:10.15252/msb.202010105)
Supplement: Supplementary file 6 — Source Data for Figure 5 [file MSB-17-e10105-s004.zip › Figure5A_sourcedata/GSEA_3017/nosigdb_stateD.GseaPreranked.1622795010397/DOROSZKO_U3180MG_CLUSTER_3.html]

Details for gene set DOROSZKO\_U3180MG\_CLUSTER\_3[GSEA]

|  || Dataset | state23017 |
| Phenotype | NoPhenotypeAvailable |
| Upregulated in class | na\_neg |
| GeneSet | DOROSZKO\_U3180MG\_CLUSTER\_3 |
| Enrichment Score (ES) | -0.5024679 |
| Normalized Enrichment Score (NES) | -2.990629 |
| Nominal p-value | 0.0 |
| FDR q-value | 0.0 |
| FWER p-Value | 0.0 |
Table: GSEA Results Summary

  

Fig 1: Enrichment plot: DOROSZKO\_U3180MG\_CLUSTER\_3      
 Profile of the Running ES Score & Positions of GeneSet Members on the Rank Ordered List

  

| PROBE | GENE SYMBOL | GENE\_TITLE | RANK IN GENE LIST | RANK METRIC SCORE | RUNNING ES | CORE ENRICHMENT || 1 | SMS |  |  | 45 | 0.358 | -0.0381 | No |
| 2 | HMGB3 |  |  | 80 | 0.311 | -0.0666 | No |
| 3 | BIRC5 |  |  | 130 | 0.271 | -0.1092 | No |
| 4 | MKI67 |  |  | 206 | -0.254 | -0.1758 | No |
| 5 | MCM2 |  |  | 226 | -0.256 | -0.1909 | No |
| 6 | PXMP2 |  |  | 233 | -0.257 | -0.1942 | No |
| 7 | FAF1 |  |  | 246 | -0.259 | -0.2029 | No |
| 8 | MTHFD1 |  |  | 281 | -0.262 | -0.2318 | No |
| 9 | RBL1 |  |  | 285 | -0.262 | -0.2322 | No |
| 10 | TRIP13 |  |  | 294 | -0.263 | -0.2372 | No |
| 11 | TCTEX1D2 |  |  | 297 | -0.263 | -0.2367 | No |
| 12 | POLA2 |  |  | 312 | -0.264 | -0.2472 | No |
| 13 | PSMB9 |  |  | 341 | -0.267 | -0.2705 | No |
| 14 | EXOSC8 |  |  | 347 | -0.268 | -0.2727 | No |
| 15 | RNASEH2B |  |  | 370 | -0.270 | -0.2905 | No |
| 16 | CARHSP1 |  |  | 405 | -0.274 | -0.3193 | No |
| 17 | TUBB |  |  | 410 | -0.274 | -0.3205 | No |
| 18 | MASTL |  |  | 414 | -0.275 | -0.3208 | No |
| 19 | CEP57 |  |  | 418 | -0.275 | -0.3211 | No |
| 20 | CHCHD3 |  |  | 420 | -0.276 | -0.3196 | No |
| 21 | CSE1L |  |  | 421 | -0.276 | -0.3171 | No |
| 22 | LY6E |  |  | 437 | -0.278 | -0.3284 | No |
| 23 | UHRF1 |  |  | 438 | -0.278 | -0.3260 | No |
| 24 | VOPP1 |  |  | 441 | -0.279 | -0.3253 | No |
| 25 | RAB23 |  |  | 442 | -0.279 | -0.3228 | No |
| 26 | TEDC1 |  |  | 462 | -0.281 | -0.3378 | No |
| 27 | FOXM1 |  |  | 478 | -0.282 | -0.3490 | No |
| 28 | PRADC1 |  |  | 497 | -0.284 | -0.3630 | No |
| 29 | MCM10 |  |  | 515 | -0.287 | -0.3761 | No |
| 30 | E2F1 |  |  | 521 | -0.287 | -0.3781 | No |
| 31 | WDR76 |  |  | 557 | -0.292 | -0.4076 | No |
| 32 | WDHD1 |  |  | 559 | -0.292 | -0.4060 | No |
| 33 | NUP85 |  |  | 562 | -0.292 | -0.4052 | No |
| 34 | SIVA1 |  |  | 567 | -0.293 | -0.4063 | No |
| 35 | RFC5 |  |  | 568 | -0.293 | -0.4036 | No |
| 36 | SLBP |  |  | 573 | -0.295 | -0.4047 | No |
| 37 | RFC4 |  |  | 575 | -0.295 | -0.4030 | No |
| 38 | BTG3 |  |  | 581 | -0.296 | -0.4049 | No |
| 39 | TEX30 |  |  | 592 | -0.298 | -0.4115 | No |
| 40 | DSCC1 |  |  | 596 | -0.299 | -0.4116 | No |
| 41 | RFC2 |  |  | 607 | -0.302 | -0.4180 | No |
| 42 | NCAPH2 |  |  | 616 | -0.303 | -0.4227 | No |
| 43 | MCM3 |  |  | 625 | -0.305 | -0.4273 | No |
| 44 | DCK |  |  | 631 | -0.307 | -0.4292 | No |
| 45 | ACADM |  |  | 636 | -0.308 | -0.4301 | No |
| 46 | PRC1 |  |  | 645 | -0.310 | -0.4347 | No |
| 47 | HAUS2 |  |  | 657 | -0.311 | -0.4420 | No |
| 48 | DNMT1 |  |  | 659 | -0.312 | -0.4402 | No |
| 49 | PHGDH |  |  | 662 | -0.312 | -0.4392 | No |
| 50 | HPF1 |  |  | 666 | -0.313 | -0.4392 | No |
| 51 | RAD51C |  |  | 670 | -0.313 | -0.4392 | No |
| 52 | RAD21 |  |  | 674 | -0.314 | -0.4391 | No |
| 53 | HAUS8 |  |  | 678 | -0.315 | -0.4391 | No |
| 54 | CHAF1A |  |  | 679 | -0.315 | -0.4363 | No |
| 55 | SUZ12 |  |  | 685 | -0.316 | -0.4380 | No |
| 56 | TMPO |  |  | 688 | -0.317 | -0.4370 | No |
| 57 | KIF4A |  |  | 706 | -0.320 | -0.4498 | No |
| 58 | SPC24 |  |  | 747 | -0.330 | -0.4836 | No |
| 59 | WDR34 |  |  | 752 | -0.331 | -0.4843 | No |
| 60 | ASRGL1 |  |  | 760 | -0.333 | -0.4878 | No |
| 61 | PRIM2 |  |  | 764 | -0.336 | -0.4875 | No |
| 62 | SLF1 |  |  | 765 | -0.336 | -0.4845 | No |
| 63 | UBR7 |  |  | 767 | -0.336 | -0.4825 | No |
| 64 | GINS1 |  |  | 780 | -0.338 | -0.4905 | No |
| 65 | NFIC |  |  | 781 | -0.339 | -0.4874 | No |
| 66 | MCM4 |  |  | 783 | -0.339 | -0.4853 | No |
| 67 | PKMYT1 |  |  | 795 | -0.342 | -0.4924 | No |
| 68 | FANCI |  |  | 801 | -0.345 | -0.4939 | No |
| 69 | BRCA2 |  |  | 808 | -0.347 | -0.4963 | No |
| 70 | H2AFX |  |  | 810 | -0.347 | -0.4942 | No |
| 71 | IKBIP |  |  | 813 | -0.348 | -0.4929 | No |
| 72 | CENPM |  |  | 818 | -0.349 | -0.4935 | No |
| 73 | MAD2L1 |  |  | 819 | -0.350 | -0.4903 | No |
| 74 | GINS4 |  |  | 824 | -0.351 | -0.4909 | No |
| 75 | BUB3 |  |  | 826 | -0.351 | -0.4887 | No |
| 76 | CBX5 |  |  | 842 | -0.356 | -0.4993 | Yes |
| 77 | SHCBP1 |  |  | 844 | -0.356 | -0.4970 | Yes |
| 78 | ABHD3 |  |  | 848 | -0.357 | -0.4966 | Yes |
| 79 | CENPJ |  |  | 849 | -0.357 | -0.4934 | Yes |
| 80 | RFC3 |  |  | 852 | -0.358 | -0.4921 | Yes |
| 81 | ARL6IP6 |  |  | 854 | -0.358 | -0.4898 | Yes |
| 82 | TFDP1 |  |  | 860 | -0.359 | -0.4912 | Yes |
| 83 | CDCA4 |  |  | 870 | -0.363 | -0.4962 | Yes |
| 84 | PRKDC |  |  | 871 | -0.363 | -0.4930 | Yes |
| 85 | FAM161A |  |  | 880 | -0.366 | -0.4971 | Yes |
| 86 | CCDC15 |  |  | 883 | -0.367 | -0.4957 | Yes |
| 87 | CENPQ |  |  | 884 | -0.367 | -0.4924 | Yes |
| 88 | SMC1A |  |  | 885 | -0.367 | -0.4891 | Yes |
| 89 | TCF19 |  |  | 890 | -0.369 | -0.4895 | Yes |
| 90 | PIGX |  |  | 894 | -0.371 | -0.4890 | Yes |
| 91 | KDELC2 |  |  | 897 | -0.372 | -0.4875 | Yes |
| 92 | RAD18 |  |  | 898 | -0.374 | -0.4842 | Yes |
| 93 | CKAP2 |  |  | 900 | -0.375 | -0.4817 | Yes |
| 94 | TK1 |  |  | 902 | -0.375 | -0.4793 | Yes |
| 95 | FANCA |  |  | 910 | -0.378 | -0.4824 | Yes |
| 96 | PARP2 |  |  | 912 | -0.379 | -0.4799 | Yes |
| 97 | TUBB4B |  |  | 916 | -0.380 | -0.4793 | Yes |
| 98 | TUBG1 |  |  | 917 | -0.381 | -0.4759 | Yes |
| 99 | DEPDC1B |  |  | 923 | -0.383 | -0.4771 | Yes |
| 100 | DSN1 |  |  | 926 | -0.385 | -0.4755 | Yes |
| 101 | MSH2 |  |  | 927 | -0.385 | -0.4720 | Yes |
| 102 | USP1 |  |  | 930 | -0.386 | -0.4704 | Yes |
| 103 | HAT1 |  |  | 933 | -0.387 | -0.4688 | Yes |
| 104 | PSIP1 |  |  | 934 | -0.388 | -0.4654 | Yes |
| 105 | DERA |  |  | 937 | -0.389 | -0.4638 | Yes |
| 106 | DIAPH3 |  |  | 943 | -0.390 | -0.4649 | Yes |
| 107 | RPA1 |  |  | 946 | -0.393 | -0.4632 | Yes |
| 108 | SKA1 |  |  | 950 | -0.395 | -0.4624 | Yes |
| 109 | TMEM237 |  |  | 953 | -0.396 | -0.4607 | Yes |
| 110 | AKR7A2 |  |  | 955 | -0.397 | -0.4581 | Yes |
| 111 | DTL |  |  | 956 | -0.397 | -0.4546 | Yes |
| 112 | PEG10 |  |  | 962 | -0.398 | -0.4556 | Yes |
| 113 | SKA3 |  |  | 965 | -0.399 | -0.4539 | Yes |
| 114 | HIRIP3 |  |  | 970 | -0.402 | -0.4540 | Yes |
| 115 | RTKN2 |  |  | 973 | -0.403 | -0.4523 | Yes |
| 116 | FANCD2 |  |  | 976 | -0.405 | -0.4505 | Yes |
| 117 | YEATS4 |  |  | 979 | -0.407 | -0.4487 | Yes |
| 118 | NFATC2IP |  |  | 999 | -0.413 | -0.4625 | Yes |
| 119 | CDC45 |  |  | 1000 | -0.413 | -0.4588 | Yes |
| 120 | POLD3 |  |  | 1002 | -0.415 | -0.4560 | Yes |
| 121 | RBBP7 |  |  | 1006 | -0.418 | -0.4550 | Yes |
| 122 | CDC6 |  |  | 1008 | -0.418 | -0.4522 | Yes |
| 123 | MNS1 |  |  | 1009 | -0.419 | -0.4485 | Yes |
| 124 | CCP110 |  |  | 1010 | -0.420 | -0.4448 | Yes |
| 125 | CCDC34 |  |  | 1013 | -0.421 | -0.4428 | Yes |
| 126 | CMC2 |  |  | 1017 | -0.425 | -0.4418 | Yes |
| 127 | MIS18A |  |  | 1020 | -0.427 | -0.4398 | Yes |
| 128 | GAMT |  |  | 1022 | -0.428 | -0.4370 | Yes |
| 129 | TIMELESS |  |  | 1024 | -0.429 | -0.4340 | Yes |
| 130 | SAC3D1 |  |  | 1025 | -0.429 | -0.4302 | Yes |
| 131 | NCAPG2 |  |  | 1027 | -0.430 | -0.4273 | Yes |
| 132 | TPX2 |  |  | 1034 | -0.437 | -0.4289 | Yes |
| 133 | LIG1 |  |  | 1035 | -0.437 | -0.4250 | Yes |
| 134 | CEP152 |  |  | 1040 | -0.439 | -0.4248 | Yes |
| 135 | DDB2 |  |  | 1045 | -0.443 | -0.4245 | Yes |
| 136 | KIF22 |  |  | 1051 | -0.446 | -0.4251 | Yes |
| 137 | BRIP1 |  |  | 1052 | -0.447 | -0.4212 | Yes |
| 138 | SMC2 |  |  | 1062 | -0.452 | -0.4254 | Yes |
| 139 | SCCPDH |  |  | 1063 | -0.452 | -0.4214 | Yes |
| 140 | MCM8 |  |  | 1064 | -0.452 | -0.4174 | Yes |
| 141 | EZH2 |  |  | 1070 | -0.455 | -0.4179 | Yes |
| 142 | MMS22L |  |  | 1072 | -0.457 | -0.4147 | Yes |
| 143 | DEPDC1 |  |  | 1074 | -0.457 | -0.4116 | Yes |
| 144 | PSMC3IP |  |  | 1075 | -0.457 | -0.4075 | Yes |
| 145 | MND1 |  |  | 1083 | -0.461 | -0.4098 | Yes |
| 146 | ATAD5 |  |  | 1091 | -0.465 | -0.4121 | Yes |
| 147 | ITGB3BP |  |  | 1096 | -0.467 | -0.4116 | Yes |
| 148 | ZWINT |  |  | 1099 | -0.467 | -0.4093 | Yes |
| 149 | BUB1B |  |  | 1111 | -0.477 | -0.4152 | Yes |
| 150 | RACGAP1 |  |  | 1112 | -0.479 | -0.4109 | Yes |
| 151 | MGME1 |  |  | 1113 | -0.479 | -0.4066 | Yes |
| 152 | MCUB |  |  | 1115 | -0.483 | -0.4032 | Yes |
| 153 | E2F8 |  |  | 1116 | -0.485 | -0.3989 | Yes |
| 154 | BARD1 |  |  | 1117 | -0.486 | -0.3946 | Yes |
| 155 | EMP2 |  |  | 1124 | -0.489 | -0.3957 | Yes |
| 156 | RBBP8 |  |  | 1128 | -0.494 | -0.3941 | Yes |
| 157 | NCAPD3 |  |  | 1130 | -0.496 | -0.3906 | Yes |
| 158 | ASF1B |  |  | 1133 | -0.498 | -0.3880 | Yes |
| 159 | OLFM1 |  |  | 1136 | -0.500 | -0.3854 | Yes |
| 160 | PCNA |  |  | 1142 | -0.504 | -0.3855 | Yes |
| 161 | VRK1 |  |  | 1143 | -0.505 | -0.3810 | Yes |
| 162 | IDH2 |  |  | 1146 | -0.508 | -0.3783 | Yes |
| 163 | CKAP2L |  |  | 1147 | -0.508 | -0.3737 | Yes |
| 164 | TMEM106C |  |  | 1148 | -0.509 | -0.3692 | Yes |
| 165 | HSPB11 |  |  | 1149 | -0.511 | -0.3647 | Yes |
| 166 | FAM111B |  |  | 1151 | -0.514 | -0.3610 | Yes |
| 167 | MXD3 |  |  | 1153 | -0.514 | -0.3573 | Yes |
| 168 | NUSAP1 |  |  | 1154 | -0.515 | -0.3528 | Yes |
| 169 | GMNN |  |  | 1155 | -0.516 | -0.3482 | Yes |
| 170 | CEP135 |  |  | 1156 | -0.517 | -0.3435 | Yes |
| 171 | DBF4 |  |  | 1157 | -0.520 | -0.3389 | Yes |
| 172 | MIS18BP1 |  |  | 1162 | -0.524 | -0.3379 | Yes |
| 173 | CIP2A |  |  | 1165 | -0.533 | -0.3350 | Yes |
| 174 | RRM1 |  |  | 1166 | -0.534 | -0.3303 | Yes |
| 175 | KNL1 |  |  | 1167 | -0.534 | -0.3255 | Yes |
| 176 | BRCA1 |  |  | 1171 | -0.541 | -0.3234 | Yes |
| 177 | LRRCC1 |  |  | 1173 | -0.544 | -0.3195 | Yes |
| 178 | SGO2 |  |  | 1176 | -0.546 | -0.3165 | Yes |
| 179 | ORC6 |  |  | 1179 | -0.551 | -0.3134 | Yes |
| 180 | XRCC2 |  |  | 1180 | -0.551 | -0.3085 | Yes |
| 181 | CENPU |  |  | 1182 | -0.555 | -0.3045 | Yes |
| 182 | KIF2C |  |  | 1183 | -0.558 | -0.2995 | Yes |
| 183 | CDCA3 |  |  | 1187 | -0.563 | -0.2972 | Yes |
| 184 | ASPM |  |  | 1189 | -0.567 | -0.2931 | Yes |
| 185 | FEN1 |  |  | 1191 | -0.569 | -0.2890 | Yes |
| 186 | NUF2 |  |  | 1194 | -0.574 | -0.2857 | Yes |
| 187 | DHFR |  |  | 1197 | -0.576 | -0.2824 | Yes |
| 188 | CKS1B |  |  | 1198 | -0.577 | -0.2772 | Yes |
| 189 | GTSE1 |  |  | 1210 | -0.602 | -0.2820 | Yes |
| 190 | CLSPN |  |  | 1212 | -0.605 | -0.2775 | Yes |
| 191 | KIF15 |  |  | 1213 | -0.610 | -0.2721 | Yes |
| 192 | CENPK |  |  | 1220 | -0.619 | -0.2721 | Yes |
| 193 | C21orf58 |  |  | 1222 | -0.626 | -0.2674 | Yes |
| 194 | DUT |  |  | 1223 | -0.633 | -0.2618 | Yes |
| 195 | SMC4 |  |  | 1224 | -0.638 | -0.2561 | Yes |
| 196 | MYBL2 |  |  | 1225 | -0.639 | -0.2504 | Yes |
| 197 | CKS2 |  |  | 1226 | -0.641 | -0.2447 | Yes |
| 198 | TCEAL9 |  |  | 1227 | -0.642 | -0.2390 | Yes |
| 199 | NCAPH |  |  | 1228 | -0.643 | -0.2332 | Yes |
| 200 | CDCA5 |  |  | 1244 | -0.685 | -0.2409 | Yes |
| 201 | SGO1 |  |  | 1246 | -0.691 | -0.2357 | Yes |
| 202 | AURKB |  |  | 1250 | -0.700 | -0.2322 | Yes |
| 203 | MELK |  |  | 1251 | -0.701 | -0.2260 | Yes |
| 204 | CCNA2 |  |  | 1255 | -0.716 | -0.2223 | Yes |
| 205 | UBE2T |  |  | 1256 | -0.719 | -0.2159 | Yes |
| 206 | CDCA2 |  |  | 1258 | -0.727 | -0.2104 | Yes |
| 207 | ARHGAP11A |  |  | 1262 | -0.735 | -0.2066 | Yes |
| 208 | HMGB2 |  |  | 1263 | -0.736 | -0.2000 | Yes |
| 209 | RAD51AP1 |  |  | 1264 | -0.741 | -0.1934 | Yes |
| 210 | KIF23 |  |  | 1265 | -0.741 | -0.1868 | Yes |
| 211 | KIFC1 |  |  | 1267 | -0.748 | -0.1811 | Yes |
| 212 | TACC3 |  |  | 1268 | -0.751 | -0.1744 | Yes |
| 213 | TYMS |  |  | 1272 | -0.770 | -0.1703 | Yes |
| 214 | CCNE2 |  |  | 1273 | -0.771 | -0.1634 | Yes |
| 215 | ATAD2 |  |  | 1275 | -0.775 | -0.1574 | Yes |
| 216 | NDC80 |  |  | 1278 | -0.810 | -0.1520 | Yes |
| 217 | PCLAF |  |  | 1281 | -0.828 | -0.1465 | Yes |
| 218 | ESCO2 |  |  | 1283 | -0.846 | -0.1399 | Yes |
| 219 | FAM111A |  |  | 1285 | -0.871 | -0.1330 | Yes |
| 220 | HJURP |  |  | 1290 | -0.897 | -0.1287 | Yes |
| 221 | SPC25 |  |  | 1294 | -0.932 | -0.1232 | Yes |
| 222 | RRM2 |  |  | 1295 | -0.933 | -0.1149 | Yes |
| 223 | KPNA2 |  |  | 1297 | -0.972 | -0.1071 | Yes |
| 224 | CSRP2 |  |  | 1298 | -0.986 | -0.0983 | Yes |
| 225 | FBXO5 |  |  | 1301 | -0.992 | -0.0913 | Yes |
| 226 | NCAPG |  |  | 1303 | -1.015 | -0.0832 | Yes |
| 227 | HIST1H1E |  |  | 1309 | -1.319 | -0.0760 | Yes |
| 228 | CDK1 |  |  | 1311 | -1.433 | -0.0642 | Yes |
| 229 | UBE2C |  |  | 1312 | -1.466 | -0.0511 | Yes |
| 230 | TOP2A |  |  | 1313 | -1.528 | -0.0375 | Yes |
| 231 | HIST1H1D |  |  | 1319 | -1.958 | -0.0247 | Yes |
| 232 | HIST1H4C |  |  | 1320 | -2.769 | 0.0000 | Yes |
Table: GSEA details [plain text format]

  

Fig 2: DOROSZKO\_U3180MG\_CLUSTER\_3: Random ES distribution      
 Gene set null distribution of ES for **DOROSZKO\_U3180MG\_CLUSTER\_3**
